# Tetra­aqua­bis­[4-(pyrazin-2-ylsulfanylmethyl-κ*N* ^4^)benzoato]cobalt(II)

**DOI:** 10.1107/S1600536810037906

**Published:** 2010-09-30

**Authors:** Fu-An Li

**Affiliations:** a College of Chemistry and Chemical Engineering, Pingdingshan University, Pingdingshan 467000, People’s Republic of China

## Abstract

In the title compound, [Co(C_12_H_9_N_2_O_2_S)_2_(H_2_O)_4_], the Co^II^ ion, lying on an inversion center, has an octa­hedral coordination involving two N atoms of two 4-(pyrazin-2-ylsulf­anylmeth­yl)benzoate ligands and four water mol­ecules. In the crystal, O—H⋯O hydrogen bonds between the coordinated water mol­ecules and uncoordinated carboxyl­ate O atoms, and weak π–π inter­actions [centroid–centroid distance = 4.105 (2) Å] between the benzene and pyrazine rings lead to a three-dimensional supra­molecular network.

## Related literature

For general background to the network topologies and applications of coordination polymers, see: Han *et al.* (2003 ▶); Zhao, Hong *et al.* (2002*a* ▶,*b* ▶); Zhao, Zou *et al.* (2004 ▶). For the synthesis and structure of a similar ligand, 4-(2-pyrimidinyl­thio­meth­yl)benzoic acid, see: Han *et al.* (2006 ▶).
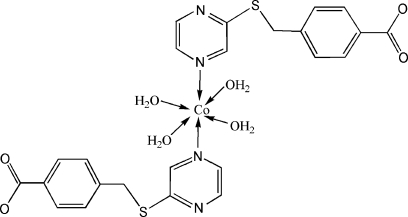


## Experimental

### 

#### Crystal data

[Co(C_12_H_9_N_2_O_2_S)_2_(H_2_O)_4_]
*M* *_r_* = 621.54
Monoclinic, 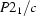

*a* = 14.6561 (11) Å
*b* = 11.0666 (8) Å
*c* = 7.9973 (6) Å
β = 90.640 (1)°
*V* = 1297.03 (17) Å^3^
*Z* = 2
Mo *K*α radiation
μ = 0.88 mm^−1^
*T* = 296 K
0.20 × 0.18 × 0.15 mm

#### Data collection

Bruker APEXII CCD diffractometer
Absorption correction: multi-scan (*SADABS*; Bruker, 2001 ▶) *T*_min_ = 0.844, *T*_max_ = 0.879
7415 measured reflections
3097 independent reflections
2131 reflections with *I* > 2σ(*I*)
*R*_int_ = 0.045

#### Refinement

*R*[*F* ^2^ > 2σ(*F* ^2^)] = 0.042
*wR*(*F* ^2^) = 0.126
*S* = 0.98
3097 reflections
178 parameters
H-atom parameters constrained
Δρ_max_ = 0.41 e Å^−3^
Δρ_min_ = −0.37 e Å^−3^

### 

Data collection: *APEX2* (Bruker, 2007 ▶); cell refinement: *SAINT* (Bruker, 2007 ▶); data reduction: *SAINT*; program(s) used to solve structure: *SHELXS97* (Sheldrick, 2008 ▶); program(s) used to refine structure: *SHELXL97* (Sheldrick, 2008 ▶); molecular graphics: *DIAMOND* (Brandenburg, 1999 ▶); software used to prepare material for publication: *SHELXTL* (Sheldrick, 2008 ▶).

## Supplementary Material

Crystal structure: contains datablocks I, global. DOI: 10.1107/S1600536810037906/hy2354sup1.cif

Structure factors: contains datablocks I. DOI: 10.1107/S1600536810037906/hy2354Isup2.hkl

Additional supplementary materials:  crystallographic information; 3D view; checkCIF report

## Figures and Tables

**Table 1:** Selected bond lengths (Å)

Co1—O1*W*	2.085 (2)
Co1—O2*W*	2.075 (2)
Co1—N1	2.169 (2)

**Table 2:** Hydrogen-bond geometry (Å, °)

*D*—H⋯*A*	*D*—H	H⋯*A*	*D*⋯*A*	*D*—H⋯*A*
O1*W*—H1*WA*⋯O2^i^	0.82	2.00	2.801 (3)	165
O1*W*—H1*WB*⋯O1^ii^	0.78	1.87	2.653 (3)	177
O2*W*—H2*WA*⋯O2^iii^	0.79	1.92	2.713 (3)	177
O2*W*—H2*WB*⋯O2^i^	0.82	1.89	2.695 (3)	167

Symmetry codes: (i) 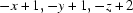; (ii) 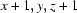; (iii) 
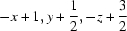
.

**Fig. 1.**
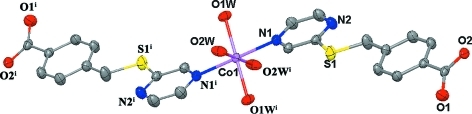
The molecular structure of the title compound. Displacement ellipsoids are drawn at the 50% probability level. [Symmetry code: (i) 2-x, 1-y, 2-z.]

**Fig. 2.**
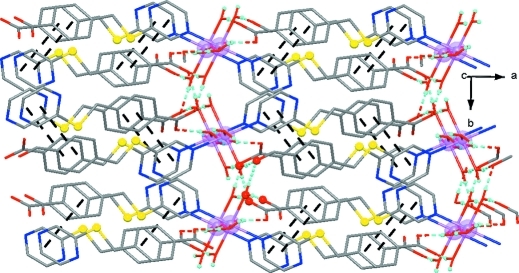
Three-dimensional supramolecular structure of the title compound. H atoms have been omitted for clarity. Dashed lines indicate hydrogen bonds and weak π–π interactions.

## supplementary crystallographic information

### Comment

Crystal engineering based on metal–organic frameworks (MOFs) using asymmetric bridging ligands as building blocks has attracted much attention owing to their potential applications as second-order nonlinear optical (NLO) materials (Han *et al.*, 2003; Zhao, Hong *et al.*, 2002*a*,b). The combination of hydrogen bonding and π–π interactions has proved to be particularly useful for the assembly of MOFs (Zhao, Zou *et al.*, 2004). Recently we have begun working on the architectures of polymeric structures containing a novel long and flexible monoanionic ligand with hybrid pyrazine and benzoate groups, namely 4-(2-pyrazinylthiomethyl)benzoic acid (Hpztmb). We report herein the synthesis and crystal structure of the title complex [Co(pztmb)_2_(H_2_O)_4_].

The title compound comprises of one Co^II^ ion, two pztmb ligands and four coordinated water molecules (Fig. 1). The Co^II^ ion lies on an inversion center in an octahedral coordination environment, with four O atoms from four coordinated water molecules in the equatorial positions and two N atoms from two different pztmb ligands in the axial sites (Table 1). The uncoordinated carboxylate O atoms and the coordinated water molecules form abundant strong hydrogen bonds (Table 2). In addition, two neighboring pztmb ligands are parallel and inversely arranged so that there is a weak π–π interaction [centroid–centroid distance = 4.105 (2) Å] between the benzene ring of one pztmb ligand and the pyrazine ring of the other one. Consequently, the hydrogen bonds and weak π–π interactions lead to a three-dimensional supramolecular network (Fig. 2).

### Experimental

The synthesis method of Hpztmb is similar to that of Hpmtmb (Han *et al.*, 2006) except that 2-mercaptopyrazine was used instead of 2-mercaptopyrimidine. A mixture of Co(NO_3_)_2_.6H_2_O (29 mg, 0.1 mmol) and Hpztmb (50 mg, 0.2 mmol) in 10 ml of H_2_O was sealed in a stainless-steel reactor with a Teflon liner and heated at 383 K for 72 h. A quantity of red single crystals were obtained after the solution was cooled to room temperature at a rate of 10 K h^-1^.

### Refinement

H atoms were positioned geometrically and refined using a riding model, with C—H = 0.93 (aromatic) and 0.97 (CH_2_) Å and *U*_iso_(H) = 1.2*U*_eq_(C). Water H atoms were found in difference Fourier maps and initially included with a tight O—H restraint [0.85 (1) Å]. In the final refinement, the positions of the water H atoms were fixed, with *U*_iso_(H) = 1.5*U*_eq_(O).

### Crystal data

**Table:**

[Co(C_12_H_9_N_2_O_2_S)_2_(H_2_O)_4_]	*F*(000) = 642
*M**_r_* = 621.54	*D*_x_ = 1.591 Mg m^−^^3^*D*_m_ = 1.591 Mg m^−^^3^*D*_m_ measured by not measured
Monoclinic, *P*2_1_/*c*	Mo *K*α radiation, λ = 0.71073 Å
Hall symbol: -P 2ybc	Cell parameters from 1622 reflections
*a* = 14.6561 (11) Å	θ = 3.1–25.8°
*b* = 11.0666 (8) Å	µ = 0.88 mm^−^^1^
*c* = 7.9973 (6) Å	*T* = 296 K
β = 90.640 (1)°	Block, red
*V* = 1297.03 (17) Å^3^	0.20 × 0.18 × 0.15 mm
*Z* = 2	

### Data collection

**Table:**

Bruker APEXII CCD diffractometer	3097 independent reflections
Radiation source: fine-focus sealed tube	2131 reflections with *I* > 2σ(*I*)
graphite	*R*_int_ = 0.045
φ and ω scans	θ_max_ = 27.9°, θ_min_ = 1.4°
Absorption correction: multi-scan (*SADABS*; Bruker, 2001)	*h* = −19→18
*T*_min_ = 0.844, *T*_max_ = 0.879	*k* = −14→10
7415 measured reflections	*l* = −5→10

### Refinement

**Table:**

Refinement on *F*^2^	Primary atom site location: structure-invariant direct methods
Least-squares matrix: full	Secondary atom site location: difference Fourier map
*R*[*F*^2^ > 2σ(*F*^2^)] = 0.042	Hydrogen site location: inferred from neighbouring sites
*wR*(*F*^2^) = 0.126	H-atom parameters constrained
*S* = 0.98	*w* = 1/[σ^2^(*F*_o_^2^) + (0.0691*P*)^2^] where *P* = (*F*_o_^2^ + 2*F*_c_^2^)/3
3097 reflections	(Δ/σ)_max_ < 0.001
178 parameters	Δρ_max_ = 0.41 e Å^−^^3^
0 restraints	Δρ_min_ = −0.36 e Å^−^^3^

### Fractional atomic coordinates and isotropic or equivalent isotropic displacement parameters (Å^2^)

**Table:**

	*x*	*y*	*z*	*U*_iso_*/*U*_eq_	
Co1	1.0000	0.5000	1.0000	0.02141 (16)	
O1	0.17691 (15)	0.4653 (2)	0.4237 (3)	0.0410 (6)	
O2	0.11201 (13)	0.3613 (2)	0.6281 (2)	0.0306 (5)	
O1W	1.01980 (14)	0.4766 (2)	1.2565 (2)	0.0342 (5)	
H1WA	0.9824	0.5172	1.3073	0.051*	
H1WB	1.0659	0.4712	1.3069	0.051*	
O2W	0.94265 (16)	0.66681 (19)	1.0537 (3)	0.0388 (6)	
H2WB	0.9184	0.6641	1.1455	0.058*	
H2WA	0.9283	0.7237	0.9993	0.058*	
N1	0.86650 (16)	0.4200 (2)	1.0353 (3)	0.0263 (5)	
N2	0.70190 (17)	0.2923 (2)	1.0414 (3)	0.0343 (6)	
C1	0.79056 (19)	0.4640 (3)	0.9652 (4)	0.0254 (6)	
H1	0.7924	0.5392	0.9134	0.030*	
C2	0.70864 (19)	0.4004 (3)	0.9674 (3)	0.0262 (6)	
C3	0.7784 (2)	0.2507 (3)	1.1130 (4)	0.0373 (8)	
H3	0.7764	0.1763	1.1669	0.045*	
C4	0.8597 (2)	0.3120 (3)	1.1112 (4)	0.0347 (8)	
H4	0.9108	0.2784	1.1631	0.042*	
C5	0.5369 (2)	0.3367 (3)	0.8688 (5)	0.0406 (9)	
H5A	0.5643	0.2679	0.8136	0.049*	
H5B	0.5274	0.3149	0.9848	0.049*	
C6	0.44575 (19)	0.3625 (3)	0.7878 (4)	0.0278 (7)	
C7	0.4284 (2)	0.4607 (3)	0.6838 (4)	0.0313 (7)	
H7	0.4745	0.5161	0.6629	0.038*	
C8	0.3426 (2)	0.4770 (3)	0.6107 (4)	0.0284 (7)	
H8	0.3324	0.5422	0.5396	0.034*	
C9	0.27219 (18)	0.3965 (3)	0.6431 (3)	0.0237 (6)	
C10	0.2892 (2)	0.2995 (3)	0.7504 (4)	0.0300 (7)	
H10	0.2424	0.2458	0.7752	0.036*	
C11	0.3750 (2)	0.2825 (3)	0.8202 (4)	0.0315 (7)	
H11	0.3855	0.2166	0.8898	0.038*	
C12	0.18071 (18)	0.4100 (3)	0.5590 (3)	0.0254 (6)	
S1	0.61446 (5)	0.46202 (8)	0.86052 (11)	0.0350 (2)	

### Atomic displacement parameters (Å^2^)

**Table:**

	*U*^11^	*U*^22^	*U*^33^	*U*^12^	*U*^13^	*U*^23^
Co1	0.0161 (3)	0.0262 (3)	0.0219 (3)	0.0013 (2)	−0.00308 (19)	0.0018 (2)
O1	0.0273 (12)	0.0612 (17)	0.0342 (12)	−0.0043 (11)	−0.0104 (10)	0.0150 (11)
O2	0.0206 (11)	0.0422 (13)	0.0291 (11)	−0.0056 (9)	0.0006 (9)	−0.0023 (9)
O1W	0.0243 (11)	0.0539 (16)	0.0241 (10)	0.0068 (10)	−0.0071 (9)	−0.0008 (9)
O2W	0.0532 (15)	0.0305 (13)	0.0330 (12)	0.0129 (11)	0.0120 (11)	0.0073 (10)
N1	0.0198 (12)	0.0315 (15)	0.0274 (12)	−0.0017 (10)	−0.0027 (10)	0.0012 (11)
N2	0.0242 (14)	0.0395 (17)	0.0393 (16)	−0.0048 (11)	−0.0039 (12)	0.0096 (12)
C1	0.0221 (15)	0.0297 (16)	0.0243 (14)	0.0000 (11)	−0.0029 (11)	0.0007 (12)
C2	0.0205 (14)	0.0357 (18)	0.0222 (14)	0.0002 (12)	−0.0040 (11)	−0.0019 (12)
C3	0.0318 (17)	0.039 (2)	0.0412 (19)	−0.0025 (14)	−0.0051 (14)	0.0144 (15)
C4	0.0247 (16)	0.040 (2)	0.0389 (18)	0.0008 (14)	−0.0057 (13)	0.0107 (15)
C5	0.0238 (17)	0.044 (2)	0.054 (2)	−0.0065 (14)	−0.0137 (15)	0.0147 (16)
C6	0.0206 (14)	0.0349 (18)	0.0277 (15)	−0.0008 (12)	−0.0034 (12)	0.0019 (13)
C7	0.0205 (15)	0.0365 (18)	0.0369 (17)	−0.0060 (13)	−0.0056 (12)	0.0076 (14)
C8	0.0262 (15)	0.0307 (19)	0.0281 (15)	−0.0023 (12)	−0.0041 (12)	0.0053 (12)
C9	0.0190 (14)	0.0294 (17)	0.0225 (14)	0.0012 (11)	−0.0025 (11)	−0.0040 (12)
C10	0.0203 (15)	0.0338 (18)	0.0358 (17)	−0.0057 (12)	−0.0020 (12)	0.0043 (13)
C11	0.0247 (15)	0.0320 (18)	0.0376 (17)	−0.0008 (13)	−0.0058 (13)	0.0102 (14)
C12	0.0189 (14)	0.0314 (17)	0.0257 (15)	−0.0010 (12)	−0.0031 (11)	−0.0074 (12)
S1	0.0230 (4)	0.0354 (5)	0.0464 (5)	−0.0027 (3)	−0.0123 (3)	0.0062 (4)

### Geometric parameters (Å, °)

**Table:**

Co1—O1W	2.085 (2)	C3—H3	0.9300
Co1—O2W	2.075 (2)	C4—H4	0.9300
Co1—N1	2.169 (2)	C5—C6	1.505 (4)
O1—C12	1.244 (4)	C5—S1	1.795 (3)
O2—C12	1.274 (3)	C5—H5A	0.9700
O1W—H1WA	0.8200	C5—H5B	0.9700
O1W—H1WB	0.7849	C6—C7	1.390 (4)
O2W—H2WB	0.8200	C6—C11	1.390 (4)
O2W—H2WA	0.7924	C7—C8	1.392 (4)
N1—C1	1.333 (4)	C7—H7	0.9300
N1—C4	1.345 (4)	C8—C9	1.390 (4)
N2—C3	1.334 (4)	C8—H8	0.9300
N2—C2	1.339 (4)	C9—C10	1.395 (4)
C1—C2	1.392 (4)	C9—C12	1.501 (4)
C1—H1	0.9300	C10—C11	1.383 (4)
C2—S1	1.753 (3)	C10—H10	0.9300
C3—C4	1.371 (4)	C11—H11	0.9300
			
O2W—Co1—O2W^i^	180.000 (1)	C4—C3—H3	118.3
O2W—Co1—O1W	87.66 (9)	N1—C4—C3	120.9 (3)
O2W^i^—Co1—O1W	92.34 (9)	N1—C4—H4	119.6
O2W—Co1—O1W^i^	92.34 (9)	C3—C4—H4	119.6
O2W^i^—Co1—O1W^i^	87.66 (9)	C6—C5—S1	113.4 (2)
O1W—Co1—O1W^i^	180.000 (1)	C6—C5—H5A	108.9
O2W—Co1—N1^i^	91.83 (9)	S1—C5—H5A	108.9
O2W^i^—Co1—N1^i^	88.17 (9)	C6—C5—H5B	108.9
O1W—Co1—N1^i^	93.62 (9)	S1—C5—H5B	108.9
O1W^i^—Co1—N1^i^	86.38 (9)	H5A—C5—H5B	107.7
O2W—Co1—N1	88.17 (9)	C7—C6—C11	118.5 (3)
O2W^i^—Co1—N1	91.83 (9)	C7—C6—C5	124.2 (3)
O1W—Co1—N1	86.38 (9)	C11—C6—C5	117.3 (3)
O1W^i^—Co1—N1	93.62 (9)	C6—C7—C8	120.8 (3)
N1^i^—Co1—N1	180.00 (13)	C6—C7—H7	119.6
Co1—O1W—H1WA	109.5	C8—C7—H7	119.6
Co1—O1W—H1WB	128.6	C9—C8—C7	120.5 (3)
H1WA—O1W—H1WB	111.3	C9—C8—H8	119.8
Co1—O2W—H2WB	109.5	C7—C8—H8	119.8
Co1—O2W—H2WA	134.4	C8—C9—C10	118.7 (3)
H2WB—O2W—H2WA	113.9	C8—C9—C12	120.9 (3)
C1—N1—C4	116.6 (3)	C10—C9—C12	120.3 (3)
C1—N1—Co1	123.1 (2)	C11—C10—C9	120.5 (3)
C4—N1—Co1	119.6 (2)	C11—C10—H10	119.7
C3—N2—C2	115.6 (3)	C9—C10—H10	119.7
N1—C1—C2	121.8 (3)	C10—C11—C6	121.0 (3)
N1—C1—H1	119.1	C10—C11—H11	119.5
C2—C1—H1	119.1	C6—C11—H11	119.5
N2—C2—C1	121.7 (3)	O1—C12—O2	123.9 (3)
N2—C2—S1	120.1 (2)	O1—C12—C9	118.0 (3)
C1—C2—S1	118.2 (2)	O2—C12—C9	118.1 (3)
N2—C3—C4	123.4 (3)	C2—S1—C5	100.22 (15)
N2—C3—H3	118.3		
			
O2W—Co1—N1—C1	48.1 (2)	S1—C5—C6—C11	−165.9 (2)
O2W^i^—Co1—N1—C1	−131.9 (2)	C11—C6—C7—C8	−1.5 (5)
O1W—Co1—N1—C1	135.9 (2)	C5—C6—C7—C8	178.6 (3)
O1W^i^—Co1—N1—C1	−44.1 (2)	C6—C7—C8—C9	1.3 (5)
O2W—Co1—N1—C4	−141.6 (2)	C7—C8—C9—C10	0.1 (4)
O2W^i^—Co1—N1—C4	38.4 (2)	C7—C8—C9—C12	−177.2 (3)
O1W—Co1—N1—C4	−53.8 (2)	C8—C9—C10—C11	−1.4 (4)
O1W^i^—Co1—N1—C4	126.2 (2)	C12—C9—C10—C11	176.0 (3)
C4—N1—C1—C2	−1.2 (4)	C9—C10—C11—C6	1.2 (5)
Co1—N1—C1—C2	169.4 (2)	C7—C6—C11—C10	0.3 (5)
C3—N2—C2—C1	0.4 (4)	C5—C6—C11—C10	−179.8 (3)
C3—N2—C2—S1	177.1 (2)	C8—C9—C12—O1	23.7 (4)
N1—C1—C2—N2	0.6 (5)	C10—C9—C12—O1	−153.6 (3)
N1—C1—C2—S1	−176.1 (2)	C8—C9—C12—O2	−158.2 (3)
C2—N2—C3—C4	−0.7 (5)	C10—C9—C12—O2	24.5 (4)
C1—N1—C4—C3	0.9 (5)	N2—C2—S1—C5	−6.9 (3)
Co1—N1—C4—C3	−170.1 (3)	C1—C2—S1—C5	169.9 (2)
N2—C3—C4—N1	0.1 (5)	C6—C5—S1—C2	178.9 (2)
S1—C5—C6—C7	14.0 (4)		

Symmetry codes: (i) −*x*+2, −*y*+1, −*z*+2.

### Hydrogen-bond geometry (Å, °)

**Table:**

*D*—H···*A*	*D*—H	H···*A*	*D*···*A*	*D*—H···*A*
O1W—H1WA···O2^ii^	0.82	2.00	2.801 (3)	165
O1W—H1WB···O1^iii^	0.78	1.87	2.653 (3)	177
O2W—H2WA···O2^iv^	0.79	1.92	2.713 (3)	177
O2W—H2WB···O2^ii^	0.82	1.89	2.695 (3)	167

Symmetry codes: (ii) −*x*+1, −*y*+1, −*z*+2; (iii) *x*+1, *y*, *z*+1; (iv) −*x*+1, *y*+1/2, −*z*+3/2.
